# An Antibiotic-Impacted Microbiota Compromises the Development of Colonic Regulatory T Cells and Predisposes to Dysregulated Immune Responses

**DOI:** 10.1128/mBio.03335-20

**Published:** 2021-02-02

**Authors:** Xiaozhou Zhang, Timothy C. Borbet, Angela Fallegger, Matthew F. Wipperman, Martin J. Blaser, Anne Müller

**Affiliations:** a Institute of Molecular Cancer Research, University of Zurich, Zurich, Switzerland; b Department of Pathology, New York University School of Medicine, New York, New York, USA; c Immunology Program, Sloan Kettering Institute, New York, USA; d Clinical and Translational Science Center, Weill Cornell Medicine, New York, New York, USA; e Center for Advanced Biotechnology and Medicine, Rutgers University, Piscataway, New Jersey, USA; University of Maryland School of Medicine

**Keywords:** CD4 T cells, Treg deficiency, broad-spectrum antibiotics, colonic regulatory T cells, dysregulated Th1 responses, dysregulated immune response, metabolic disorders, microbiota, transmaternal exposure

## Abstract

Antibiotic exposure early in life and other practices impacting the vertical transmission and ordered assembly of a diverse and balanced gut microbiota are associated with a higher risk of immunological and metabolic disorders such as asthma and allergy, autoimmunity, obesity, and susceptibility to opportunistic infections. In this study, we used a model of perinatal exposure to the broad-spectrum antibiotic ampicillin to examine how the acquisition of a dysbiotic microbiota affects neonatal immune system development. We found that the resultant dysbiosis imprints in a manner that is irreversible after weaning, leading to specific and selective alteration of the colonic CD4^+^ T-cell compartment. In contrast, colonic granulocyte and myeloid lineages and other mucosal T-cell compartments are unaffected. Among colonic CD4^+^ T cells, we observed the most pronounced effects on neuropilin-negative, RORγt- and Foxp3-positive regulatory T cells, which are largely absent in antibiotic-exposed mice even as they reach adulthood. Immunomagnetically isolated dendritic cells from antibiotic-exposed mice fail to support the generation of Foxp3^+^ regulatory T cells (Tregs) from naive T cells *ex vivo*. The perinatally acquired dysbiotic microbiota predisposes to dysregulated effector T-cell responses to Citrobacter rodentium or ovalbumin challenge. The transfer of the antibiotic-impacted, but not healthy, fecal microbiota into germfree recipients recapitulates the selective loss of colonic neuropilin-negative, RORγt- and Foxp3-positive Tregs. The combined data indicate that the early-life acquisition of a dysbiotic microbiota has detrimental effects on the diversity and microbial community composition of offspring that persist into adulthood and predisposes to inappropriate T-cell responses that are linked to compromised immune tolerance.

## Abstract (precis)

The assembly of microbial communities that populate all mucosal surfaces of the human body begins right after birth. This process is prone to disruption as newborns and young infants are increasingly exposed to antibiotics, both deliberately for therapeutic purposes, and as a consequence of transmaternal exposure.

## INTRODUCTION

The lifelong process of the colonization of environmentally exposed surfaces of mammals—such as the skin, mouth, gut and vagina—begins at birth, with the infant acquiring its primordial microbiota from the mother (1–5). Of all the microbially colonized niches of the body, the lower gastrointestinal tract harbors by far the most abundant community of resident microbes, and by adulthood, it is colonized by thousands of species and on the order of 100 trillion microbial cells (6–9). These microbes provide numerous benefits to their host, including the processing of otherwise indigestible compounds, production of nutrients and vitamins, defense against pathogens, and education of the mucosal immune system (3–5, 10–13). Diverse molecular interactions between resident microbes and their mucosal niche direct the development of immune cell populations and immune responses, and these, in turn, help shape the composition of the microbiota (11, 14–16).

Early life is the critical period for the development and assembly of the mature microbiome in humans and mice (16–20). The dysbiosis of resident microbes has been linked to both immunological and metabolic disorders, including allergy, autoimmunity, inflammatory bowel disease, and obesity, as well as to opportunistic infections (3, 21–24, 84, 85). Consequently, the disruption of microbial communities, or interference with their assembly and maturation in the first weeks and months of life, predisposes to a higher risk of developing asthma and other allergies (25–27), obesity and type 2 diabetes (28–30), type I diabetes (31, 32), and other manifestations of autoimmunity later in life (33) and reduces neonatal immune defenses against viral (34) and bacterial (35–37) pathogens in humans and/or experimental animals. Cesarean section delivery, formula instead of breastfeeding, and early-life exposure to antibiotics all have been linked to higher rates of such metabolic, chronic inflammatory, and immunological disorders (3, 4, 16, 20, 38, 39, 84, 85). Infants and young children may be exposed to antibiotics either directly for the purpose of treatment of respiratory, cutaneous, or gastrointestinal infection, subtherapeutically through their mothers perinatally or during breastfeeding, or through environmental exposures. In the United States, more than 40% of pregnant women receive antibiotics in labor, either for group B streptococcus (GBS) prophylaxis or to prevent postpartum infections after cesarean delivery (40, 41), and 70% of infants receive antibiotics in the first year of life (27, 39); the most commonly used antibiotics in both settings are β-lactams.

In this study, we sought to investigate whether perinatal exposure of lactating dams and their pups to the commonly prescribed broad-spectrum β-lactam antibiotic ampicillin would affect the developing neonatal immune system of the offspring, in particular at mucosal sites known to host a resident microbiota. We found that this antibiotic exposure has long-term effects on the diversity and community structure of the gastrointestinal microbiota and has strong and persistent effects on the T-cell compartment of the intestinal immune system. The observed changes could be recapitulated by fecal transplantation into germfree mice and resulted in dysregulated Th1 and Th2 immune responses to infectious and allergen challenge later in life.

## RESULTS

### Perinatal antibiotic exposure has lasting consequences for the composition and diversity of the gut microbiota of offspring.

**FIG 1:**
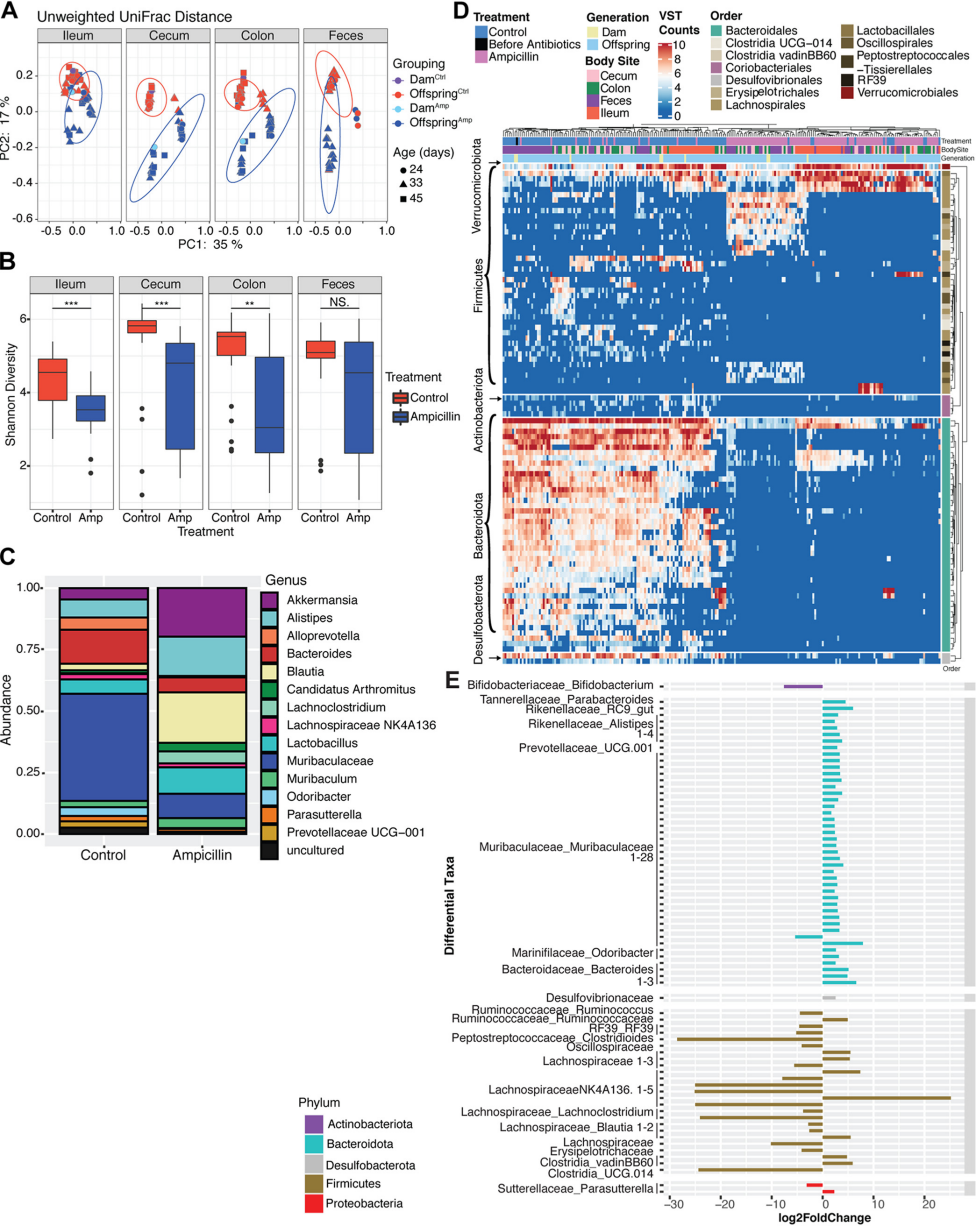
Perinatal maternal antibiotic exposure has long-lasting consequences for the gastrointestinal microbiota of offspring. Dams and their offspring were exposed to ampicillin via the drinking water from day 5 to 10 of the pups’ lives. 16S rRNA sequencing was performed on mouse intestinal tract and fecal samples. (A) Unweighted UniFrac beta diversity, rarefied at 5,000 reads, was plotted along two principal-component axes, plots separated by body site; each ellipse represents the 95% confidence interval for that treatment group. Samples are color-coded by treatment (control, red; ampicillin, blue) and generation; symbols indicate the age of the offspring mice at the specified sample collection. Adonis testing was used to determine whether there were significant differences (*P* < 0.01) between microbial communities based on treatment at each body site. (B) Alpha diversity, based on Shannon species evenness, was calculated for the offspring mice in each treatment group by body site, and statistical significance was determined by Wilcoxon rank sum test. *, *P* < 0.05; **, *P* < 0.01; ***, *P* < 0.001; NS., not significant. (C) Sequence variant counts of offspring fecal and colonic microbiota were combined within treatment groups, and relative taxonomic abundance at the genus level was determined and plotted for the 50 most abundant taxa. (D) The DESeq2 pipeline was used to generate a heatmap of the 93 significantly differentially abundant taxa (*P* < 0.01) across a total of 229 samples from 67 control or ampicillin-exposed mice (48 samples from cecum, 52 from colon, 55 from ileum, and 74 from feces). Clustering is unsupervised, and bars along the top of the plot are used to indicate treatment, body sampling site, and the mouse generation from which the samples were collected. Phyla are shown along the left side of the heat map. Taxonomy at the order level is color-coded at the right side. (E) Differentially abundant taxa in the colonic microbiota between control and ampicillin-exposed and control mice were determined using DESeq2 at the genus level. Only taxa significantly enriched in control (right side of plot) or significantly enriched in the ampicillin-exposed mice (left side of plot) at a *P* value of <0.01 are shown.

Exposure to antibiotics early in life, either directly through administration to newborn pups or via indirect, transmaternal exposure of suckling pups to antibiotics in their mothers’ milk, is known to be detrimental in adults in models of obesity, type I diabetes, allergic asthma, and bacterial infection (12, 28, 30, 31, 33, 42). To examine whether the exposure of lactating dams and pups to antibiotics affects the microbiota composition of their offspring, we administered the broad-spectrum β-lactam ampicillin to dams via the drinking water at a calculated therapeutic dose of 100 mg/kg of body weight/day when their offspring were between 5 and 10 days old. Both the offspring and their mothers were sacrificed when the offspring reached 24, 33, or 45 days of age, and DNA extracted from ileal, cecal, colonic, and fecal samples was used for microbial community analysis based on high-throughput 16S rRNA V4 region sequencing. As determined by calculating both unweighted and weighted UniFrac distances, microbial communities at all three sampling sites and in the feces differed significantly between offspring of control and ampicillin-exposed mice, and these differences persisted over time; as expected, the offspring clustered with their respective mothers without exception (Fig. 1A; see also Fig. S1A in the supplemental material) (*P* < 0.001, Adonis test). Species richness and evenness were significantly lower in offspring of ampicillin-treated mice at all three primary sampling sites and at all three time points (Fig. 1B; Fig. S1B) (*P* < 0.05, Wilcoxon rank sum test), although not in the feces. Consistent with their overall reduced diversity, offspring of ampicillin-exposed mice showed reduced amplicon sequence variants (ASVs), with very few ASVs (e.g., *Akkermansia*) being more abundant (Fig. 1C). Unsupervised hierarchical clustering of the DESeq2-derived variance stabilized transformed (vst) count data of differentially abundant ASVs between microbiota samples (false-discovery rate [FDR] < 0.01) segregated the offspring of control and ampicillin-treated dams onto two distinct branches of the taxonomic heat map, with few exceptions (Fig. 1D; Fig. S1C), with the offspring clustering with their respective mothers (Fig. 1D; Fig. S1C). Differentially abundant taxa at the genus level that were selectively lost in virtually all offspring of ampicillin-exposed dams included Muribaculum intestinale, Culturomica massiliensis, Prevotella shahii, and two *Bacteroides* species (Bacteroides rodentium and B. caecimuris) (Fig. 1E). Clostridial species, including Clostridium aldenense, C. indolis, and C. amygdalinum, bloomed in a subset of offspring of antibiotic-exposed dams (Fig. 1D; Fig. S1C). The combined data suggest that as few as 5 days of such antibiotic exposure during lactation profoundly alters the microbial community structure and composition of the offspring, persisting into young adulthood.

10.1128/mBio.03335-20.1 FIG S1 Alternative analyses of 16S rRNA sequencing used to evaluate gut microbial communities in mice. (A) Weighted UniFrac beta diversity plots, rarefied at 5,000 reads, were plotted and separated by body site, and each ellipse represents the 95% confidence interval for that treatment group. There were significant differences (*P* < 0.001, Adonis test) between microbial communities based on treatment at each body site. (B) Species richness was determined by calculating the observed number of ASVs in each treatment group across the body sites. Statistical testing used the Wilcoxon rank sum test. *, *P* < 0.05; **, *P* < 0.01. (C) Heat map of the 93 significantly differentially abundant taxa (*P* < 0.01) across a total of 229 samples from 67 control or ampicillin-exposed mice as shown in Fig. 1D in the main text, except that the taxonomic levels of genus and species are fully annotated (annotation is labeled as genus_species). Taxonomy at the order level is color-coded at the right side. Download FIG S1, JPG file, 1.5 MB. Copyright © 2021 Zhang et al. 2021 Zhang et al. This content is distributed under the terms of the Creative Commons Attribution 4.0 International license.

### Early-life antibiotic exposure dysregulates colonic lamina propria CD4^+^ T-cell populations.

**FIG 2:**
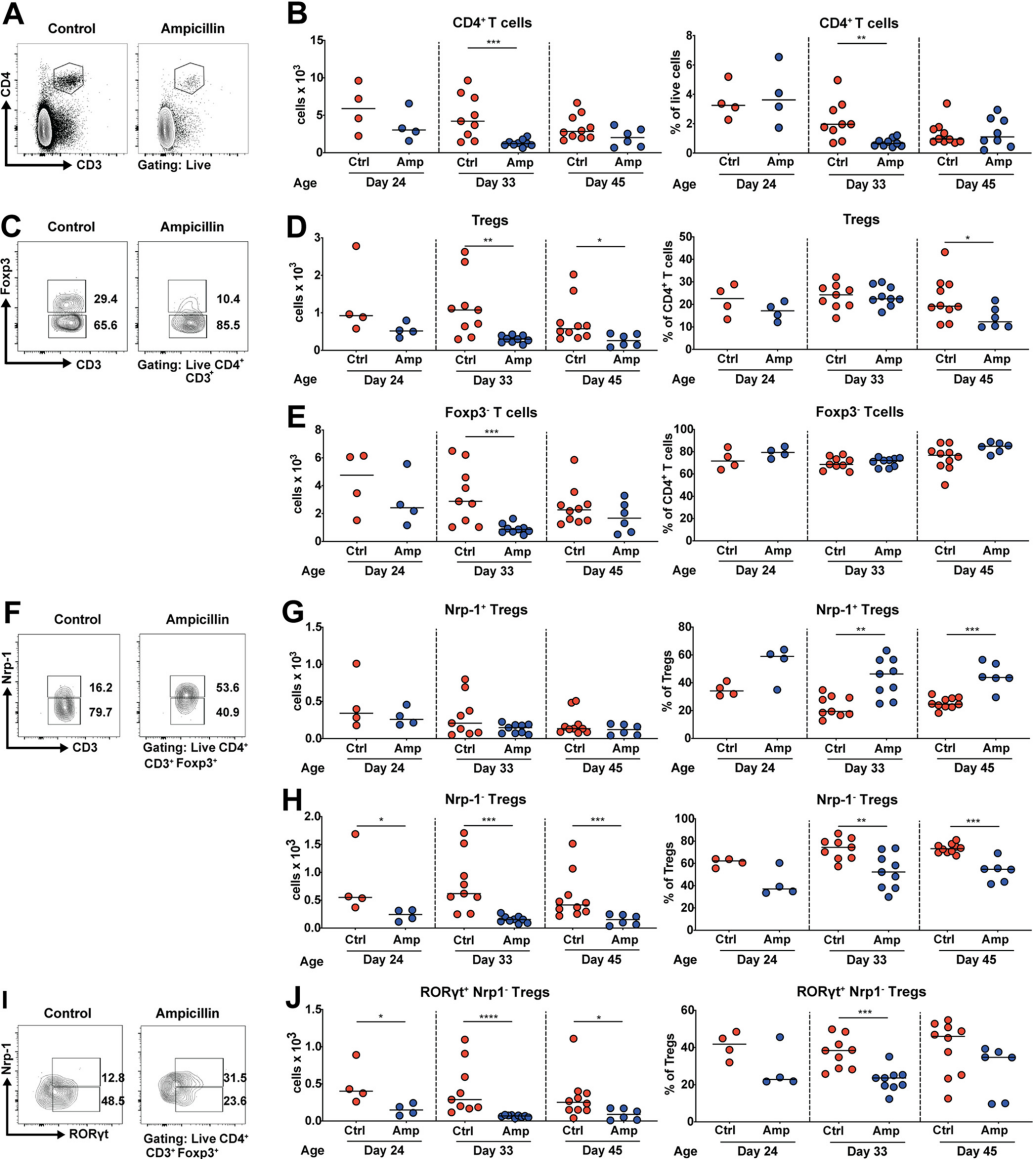
Perinatal maternal antibiotic exposure alters offspring colonic lamina propria CD4^+^ T-cell population frequencies and absolute numbers. Dams and their offspring were exposed to ampicillin via the drinking water from day 5 to 10 of the pups’ lives, and colonic lamina propria leukocyte populations of offspring were isolated at the age of 24, 33, or 45 days and analyzed by flow cytometry. (A and B) Absolute counts per organ, and frequencies among live cells, of CD4^+^ T cells in the colonic lamina propria. Representative fluorescence-activated cell sorting (FACS) plots are shown in panel A, and summary plots are shown in panel B. (C to E) Absolute counts, and frequencies among all CD4^+^ T cells, of Foxp3^+^ Tregs (D) and Foxp3^−^ effector T cells (E). Representative FACS plots are shown in panel C. (F to H) Absolute counts per organ, and frequencies among all Foxp3^+^ Tregs, of neuropilin-1-positive (Nrp-1^+^) Tregs (G) and Nrp-1^−^ Tregs (H). Representative FACS plots are shown in panel F. (I and J) Absolute counts per organ, and frequencies among all Foxp3^+^ Tregs, of RORγt^+^ Nrp-1^−^ Tregs. Representative FACS plots are shown in panel I. Two independent studies were pooled for the day 33 and 45 time points, and one study is shown for the day 24 time point. Horizontal lines indicate medians throughout; statistical analyses used the Mann-Whitney test. *, *P* < 0.05; **, *P* < 0.01; ***, *P* < 0.005; ****, *P* < 0.001. Only statistically significant differences are indicated with asterisks.

In parallel to the analysis of the gut microbiota, we systematically investigated whether, and how, such ampicillin exposure affects the developing murine immune system of the offspring. We isolated leukocytes at the same three time points as used for 16S rRNA analyses and sampled multiple lymphoid and nonlymphoid tissues, including stomach, colon, mesenteric lymph nodes (MLNs), and lung. Multidimensional flow cytometric analyses were performed on single-cell suspensions to determine the abundance and frequencies of specific myeloid populations, granulocytes and T-lymphocytes. The dominant myeloid and two major granulocyte populations of the lung, lymphoid tissues, and the gastrointestinal (GI) tract lamina propria (LP) were largely unchanged by the ampicillin exposure (as shown representatively for macrophages, monocytes, and the three major populations of dendritic cells (DCs) as well as neutrophils and eosinophils of the colonic LP in Fig. S2A). In contrast, in the ampicillin-exposed mice, CD4^+^ T cells were reduced in both number and frequency in the colonic LP relative to controls (Fig. 2A and B), as were CD8^+^ T cells (Fig. S2A). The selective reduction of T cells was specific for the colon and not observed in the gastric LP, lung, or MLNs (Fig. S2B to D). Among CD4^+^ T cells, both Foxp3^+^ regulatory T cells and Foxp3^−^ T cells were reduced in absolute numbers, reflecting the generally reduced pool of CD4^+^ T cells (Fig. 2C to E). Two subsets of regulatory T cells (Tregs) co-occur in the colonic LP and MLNs; one subset is selected in the thymus and expresses neuropilin-1 (Nrp-1), whereas the other arises in the periphery, is Nrp-1 negative, and is considered “peripherally induced” (“pTregs”) (43). Such Nrp-1^−^ Tregs represent an extremely abundant population in colons colonized by a healthy and diverse microbiota, where they comprise the majority of all Tregs (Fig. 2F to H) and approximately one-fifth of all T cells (Fig. 2B and H). Only Nrp-1^−^ Tregs were selectively absent in the colonic LP and MLNs of offspring of ampicillin-treated mice, with reductions in their absolute numbers by 60 to 80%, whereas Nrp-1^+^ Tregs were present at normal levels (Fig. 2F to H; Fig. S2D). The colonic Nrp-1^−^ Tregs that were underrepresented in the antibiotic-exposed mice express the transcription factor RORγt (Fig. 2I and J), a common property of GI tract resident Tregs (44). The selective Nrp-1^−^ RORγt^+^ Treg deficiency in the antibiotic-exposed mice persisted into early adulthood (Fig. 2F to J).

10.1128/mBio.03335-20.2 FIG S2 Perinatal antibiotic exposure has no effect on colonic lamina propria myeloid or granulocyte populations or the T-cell compartment of the gastric lamina propria and lung. (A to D) Dams and pups were exposed or not to ampicillin via the drinking water from day 5 to 10 of the pups’ lives, and colonic and gastric lamina propria, lung, and MLNs of offspring were isolated at the age of 24, 33, or 45 days and analyzed by flow cytometry. (A) Absolute counts per organ of the indicated myeloid, granulocyte, and CD4^−^ (TCRα/β^+^ CD3^+^) T-cell populations, as assessed at the age of 33 days in the colonic lamina propria. (B) Absolute counts of gastric lamina propria CD4^+^ T cells and of Foxp3^+^ Tregs, and frequencies among all Foxp3^+^ Tregs, of neuropilin-1-positive (Nrp-1^+^) Tregs and Nrp-1^−^ Tregs. (C) Absolute counts in one lung lobe of CD4^+^ T cells and Foxp3^+^ Tregs, and frequencies among all Foxp3^+^ Tregs, of Nrp-1^+^ Tregs and Nrp-1^−^ Tregs. (D) Absolute counts of MLN CD4^+^ T cells and Foxp3^+^ Tregs, and frequencies among all Foxp3^+^ Tregs, of Nrp-1^+^ Tregs and Nrp-1^−^ Tregs. (E) Dams and pups were exposed to ampicillin via the drinking water from days 5 to 10 of the pups’ lives, and their offspring were cohoused or not with age-matched controls after weaning and until the study endpoint at 45 days of age; a separate parallel group of pups was directly treated with ampicillin via daily oral gavage from days 5 to 10 of life (direct treatment). Absolute counts per organ of the indicated T-cell and Treg populations are shown. (F) Dams and pups were exposed to ampicillin via the drinking water from days 5 to 10 of the pups’ lives, and colonic lamina propria preparations were analyzed for Ki67 expression as a marker of proliferation. Representative FACS plots and a summary plot of Ki67 expression in Nrp-1^−^ Tregs is shown. Data in panel A are from one experiment. Data in panels B to D are from one study for days 24 and 33 and pooled from two studies for day 45. Cohousing data in panel E are pooled from two studies; the direct treatment group is from one study, but representative of two independently conducted ones. Data in F is from one study and representative of two independently conducted ones. Horizontal lines indicate medians throughout; statistical analyses used the Mann-Whitney test. *P < 0.05; **P < 0.01. Only statistically significant differences are indicated with asterisks. Download FIG S2, JPG file, 1.3 MB. Copyright © 2021 Zhang et al. 2021 Zhang et al. This content is distributed under the terms of the Creative Commons Attribution 4.0 International license.

Cohousing of the offspring of antibiotic-exposed mice with age-matched normal control mice for 4 weeks starting at the time of weaning failed to reverse the consequences of antibiotic exposure; rather, cohoused normal mice adopted the Treg deficiency of their dysbiotic cage mates (Fig. S2E). This finding is consistent with our previous observation that the antibiotic-perturbed dysbiosis invades, and ultimately dominates over, a healthy, diverse gastrointestinal microbiota (29). Direct (as opposed to transmaternal) exposure of newborn pups on days 5 to 10 of age to ampicillin administered at a therapeutic dose (100 mg/kg/day) had no effects on colonic Treg populations as assessed at 45 days of age (Fig. S2E).

To address whether the colonic Treg reduction in offspring of antibiotic-exposed mice is due to defects in their local expansion, we stained for Ki67 as a marker of proliferation. The vast majority of colonic Nrp-1^−^ Tregs were Ki67 positive, and the frequency of Ki67-positive Nrp-1^−^ Tregs did not differ between treatments (Fig. S2F). The combined data collected across time points and in several lymphoid and nonlymphoid tissues indicate that (i) CD4^+^ T-cell residence in the colon, but less so at other mucosal sites, requires an unperturbed microbiota and (ii) populations of Nrp-1^−^ RORγt^+^ colonic regulatory T cells are selectively and disproportionately dependent on a healthy and diverse community of intestinal microbes.

### Dendritic cells from antibiotic-pretreated donors are incapable of Treg generation *ex vivo*.

**FIG 3:**
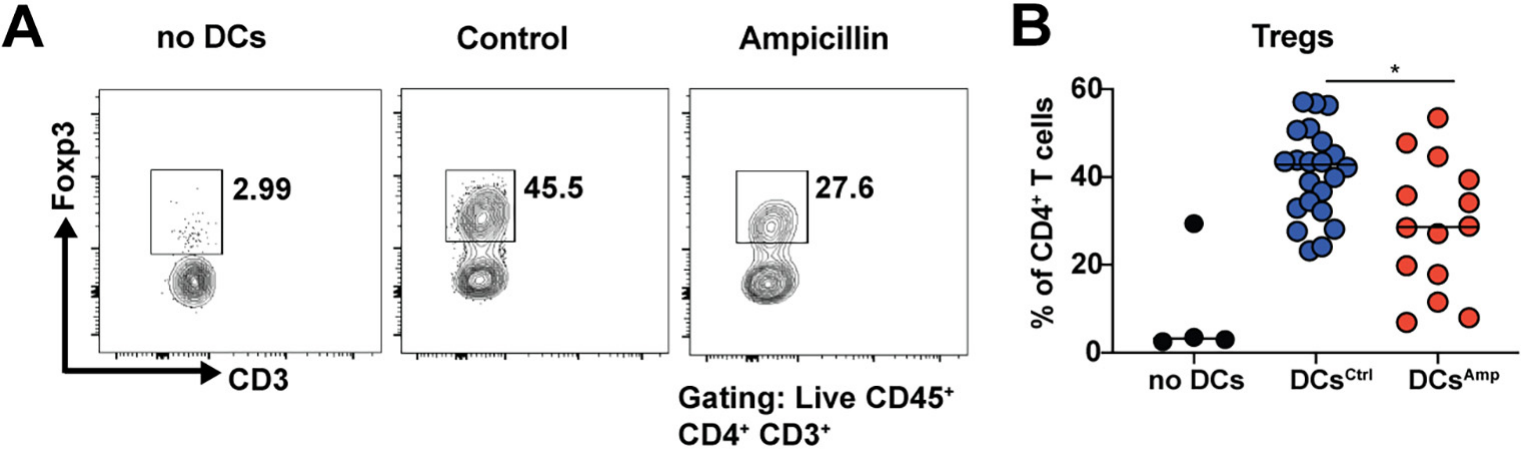
Dendritic cells isolated from the MLNs of dysbiotic offspring of antibiotic-exposed dams are less capable of inducing Foxp3 expression in cocultured naive T cells than DCs from control offspring. Immunomagnetically isolated MLN CD11c^+^ DCs from 45-day-old offspring of antibiotic-exposed dams and age-matched control offspring were cultured at 1:5 ratios with naive splenic T cells (20,000 DCs and 100,000 T cells). Cocultures were stained for CD4, and Foxp3 and Treg populations were quantified by flow cytometry. (A) Representative FACS plots of the indicated conditions. (B) Summary plot of all cocultures; data are pooled from two independent experiments. Each symbol represents one coculture; two cultures were assessed per mouse. Horizontal lines indicate medians; statistical analyses used the Mann-Whitney test. *, *P* < 0.05.

We next asked whether the selective Treg deficiency observed in the colons and MLNs of antibiotic-exposed mice is due to numerical defects or, alternatively, to functional defects of dendritic cells and their ability to promote Foxp3 expression and Treg differentiation in cocultured naive T cells. We first examined the frequencies of the dominant colonic and MLN DC populations but found no major numerical differences (Fig. S3A and B). We then immunomagnetically isolated CD11c^+^ dendritic cells from the MLNs of the antibiotic-exposed or control mice and cocultured them with naive splenic T cells, which were assessed by flow cytometry for Foxp3 expression 3 days later. Whereas dendritic cells from donors with an unperturbed microbiota induced strong Foxp3 expression in the cocultured T cells, this was markedly reduced for dendritic cells from the dysbiotic donors (Fig. 3). Since DCs migrating to the MLNs from the colonic LP provide the initial signals during the multistep process of Treg induction and intestinal tolerance development, which begins with DC-dependent Treg priming in the MLNs and is followed by Treg homing to the gut and their local expansion in the colonic LP (45), our data indicate that antibiotic-induced dysbiosis leads to Treg deficiency by compromising the tolerogenic activities of dendritic cells.

10.1128/mBio.03335-20.3 FIG S3 Perinatal antibiotic exposure has no numerical effects on colonic and MLN lamina propria DC populations. (A and B) Dams and pups were exposed or not to ampicillin via the drinking water from days 5 to 10 of the pups’ lives, and colonic lamina propria and MLNs of offspring were isolated at the age of 45 days and analyzed by flow cytometry. The frequencies of the three indicated colonic DC populations among all CD45^+^ leukocytes are shown in panel A, and the frequencies of the three major migratory and two resident DC populations, quantified based on their major histocompatibility complex class II (MHC-II), CD11c, CD11b, CD103, and CD8 expression are shown in panel B. Data are from a single study but were similar at a second (day 33) analyzed time point. Download FIG S3, TIF file, 1.4 MB. Copyright © 2021 Zhang et al. 2021 Zhang et al. This content is distributed under the terms of the Creative Commons Attribution 4.0 International license.

### The reconstitution of germfree mice with microbiota harvested from antibiotic-exposed mice recapitulates the effects on colonic CD4^+^ T cellularity and Treg populations.

**FIG 4:**
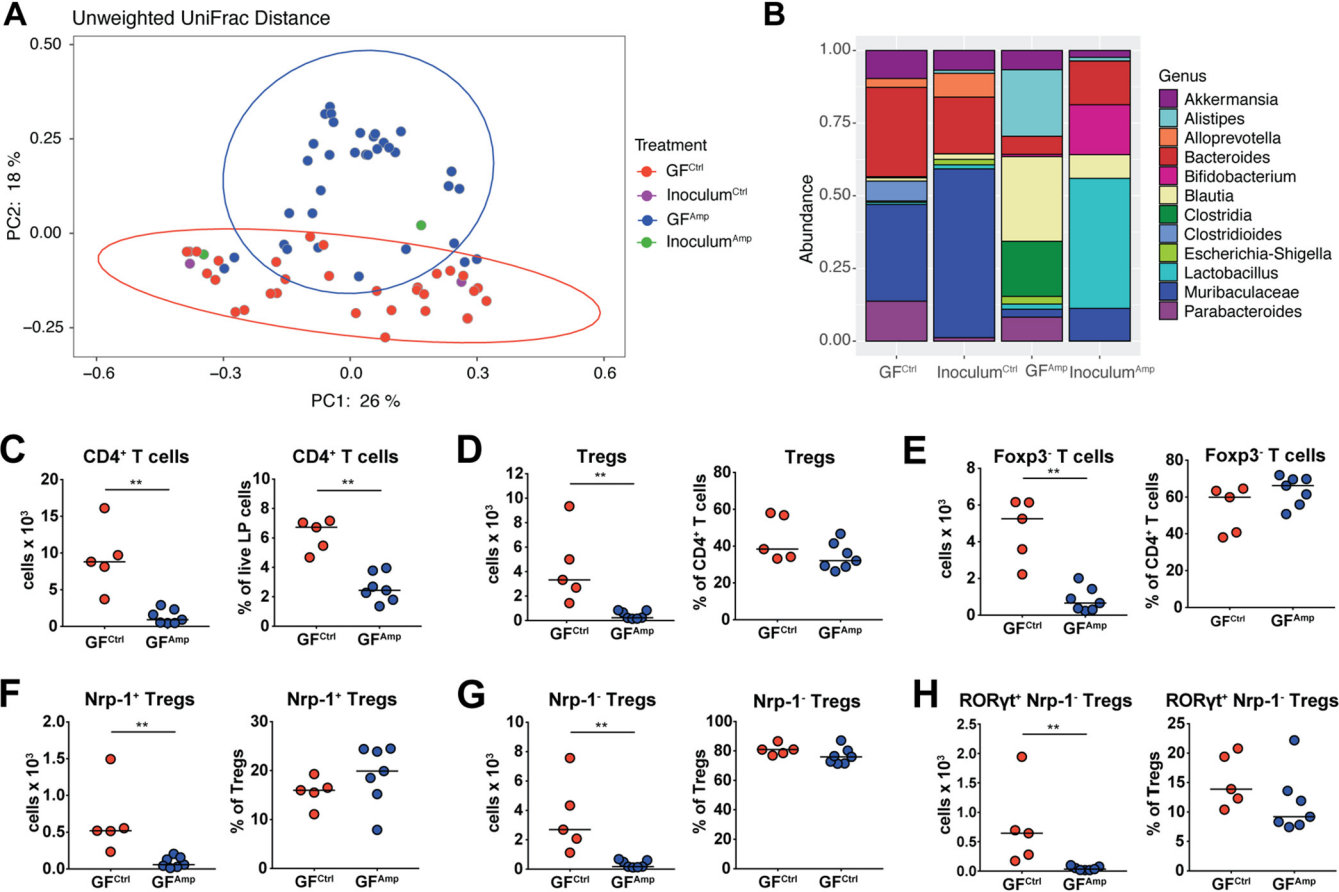
Fecal transplantation of antibiotic-perturbed microbiota into germfree mice recapitulates the colonic CD4^+^ T-cell and Treg phenotypes of the donors. Fecal microbiota from 3-week-old offspring of control or ampicillin-exposed dams (two donors each) was collected and transferred by oral gavage to 6- to 8-week-old germfree recipients. Microbial community structure and CD4^+^ T cell populations were analyzed 28 days posttransfer. (A) Unweighted UniFrac distance rarefied at 5,000 reads with the 95% confidence intervals around each recipient group; the Adonis test was used to determine statistical significance. (B) Taxonomic abundance in the now-conventionalized recipient mice compared to the fecal inocula transferred from antibiotic-perturbed or control donors. (C to H) Colonic lamina propria T-cell populations in the now-conventionalized, formerly GF mice. (C) Absolute counts per organ, and frequencies among live cells, of CD4^+^ T cells in the colonic lamina propria. (D and E) Absolute counts per organ, and frequencies among all CD4^+^ T cells, of Foxp3^+^ Tregs (D) and Foxp3^−^ effector T cells (E). (F and G) Absolute counts per organ, and frequencies among all Foxp3^+^ Tregs, of Nrp-1^+^ Tregs (F) and Nrp-1^−^ Tregs (G). (H) Absolute counts per organ, and frequencies among all Foxp3^+^ Tregs, of RORγt^+^ Nrp-1^−^ Tregs. Data in panels A and B are from two independent studies; data in panels C to H are from one study. GF^Ctrl^ and GF^Amp^, conventionalized, previously germfree recipients of control and ampicillin-impacted fecal inocula; Inoculum^Ctrl^ and Inoculum^Amp^, fecal inocula used for conventionalization. Horizontal lines indicate medians throughout; statistical analyses used the Mann-Whitney test. **, *P* < 0.01.

To determine whether the dysbiosis *per se*, rather than other physiological consequences of the ampicillin exposure, causes the selective loss of CD4^+^ T-cell populations in the colonic LP, we asked whether the perturbed microbiota could transfer the phenotypes. To determine this, we harvested fecal pellets from 3-week-old antibiotic-exposed or control pups and transferred the microbial communities into germfree recipient mice. After 4 weeks of engraftment, the recipients were analyzed with respect to their colonic microbiome relative to the microbiome of the donor mice and subjected to multidimensional flow cytometric analysis of colonic LP T-cell populations. Based on both unweighted and weighted UniFrac distance analyses, the altered microbial community structure of the donor mice was clearly recapitulated in the germfree recipients as assessed by their segregation based on treatment (Fig. 4A; Fig. S4A) (*P* < 0.001, Adonis test). The reduced diversity of the antibiotic-exposed donors was also recapitulated in the conventionalized recipient mice, with the transfer contributing to a further reduction of both the richness and the evenness of the microbiota in both groups of recipient mice, independent of donor source (Fig. 4B). Analysis of the germfree recipients also indicated that the antibiotic-induced microbiota alterations persisted over time and across experiments and hygienic conditions (Fig. S4B).

10.1128/mBio.03335-20.4 FIG S4 Fecal transplantation of antibiotic-perturbed microbiota into germfree mice has no consequences for the CD4^+^ T-cell and Treg compartments of the MLNs. Fecal microbiota from 3-week-old control or ampicillin-exposed mice (two donors each, exposed on days 5 to 10 via the dam’s drinking water) was collected and transferred by oral gavage to 6- to 8-week-old germfree recipients. Microbial community structure and the CD4^+^ T-cell populations of the MLNs were analyzed 28 days posttransfer. (A) Weighted UniFrac plot of the fecal inocula and the germfree recipient mice rarefied at 5,000 reads. (B) Weighted UniFrac plot comparing the conventional mice shown in Fig. 1 and the germfree recipient mice to assess microbial communities across experiments. Each symbol in panels A and B represents a cecal or fecal microbiota sample from one mouse. GF^Ctrl^ and GF^Amp^, conventionalized germfree recipients of control or ampicillin-pretreated fecal inoculum; Inoculum^Ctrl^ and Inoculum^Amp^, fecal inocula used for conventionalization; Dam^Ctrl^ and Dam^Amp^, antibiotic-treated or control mothers of conventionally raised offspring [Offspring^Ctrl^ and Offspring^Amp^] shown in Fig. 1). (C) Absolute counts, and frequencies among all live cells, of CD4^+^ T cells in the MLNs. (D and E) Absolute counts, and frequencies among all CD4^+^ T cells, of Foxp3^+^ Tregs (D) and Foxp3^−^ effector T cells (E). (F and G) Absolute counts, and frequencies among all Foxp3^+^ Tregs, of Nrp-1^+^ Tregs (F) and Nrp-1^−^ Tregs (G). Data in panels A and B are from two independent studies; data in panels C to G are all from one study. Horizontal lines indicate medians throughout; statistical analyses were done using the Mann-Whitney test. In none of the comparisons were the observed differences statistically significant. Download FIG S4, TIF file, 2.0 MB. Copyright © 2021 Zhang et al. 2021 Zhang et al. This content is distributed under the terms of the Creative Commons Attribution 4.0 International license.

Flow cytometric analysis of colonic LP leukocyte populations revealed that the germfree mice conventionalized with an ampicillin-perturbed microbiota had a dramatic reduction of CD4^+^ T-cell absolute numbers and frequencies among all colonic live cells (Fig. 4C), which was reflected also in the Treg and effector T-cell counts (Fig. 4D and E) and the counts of Treg subpopulations (Fig. 4F and G). In the colonic LP of the now conventionalized recipients, most Tregs were of extrathymic rather than thymic origin, as assessed by both their neuropilin-1 and RORγt expression (Fig. 4H), recapitulating the representation of the two Treg populations of mice raised under specific-pathogen-free (SPF) conditions (Fig. 2F to J). While the frequencies of Tregs among CD4^+^ T cells and of Treg subpopulations were not significantly different in the two groups of recipients, we observed that Nrp-1-positive Tregs appeared less affected by the ampicillin exposure of the donors than Nrp-1-negative RORγt-expressing Tregs (Fig. 4D to H). Analysis of the MLNs of the recipients of antibiotic-impacted microbiota revealed only modest reductions in CD4^+^ T-cell and Treg cellularity that were not statistically significant (Fig. S4C to G). Collectively, the results indicate that a healthy, diverse and unperturbed microbiota is key for the normal development of the resident colonic LP CD4^+^ T-cell population, with strong reductions observed in both regulatory and conventional CD4^+^ T cells.

### Dysbiotic mice generate excessive effector T-cell responses to bacterial pathogen or allergen challenge.

**FIG 5:**
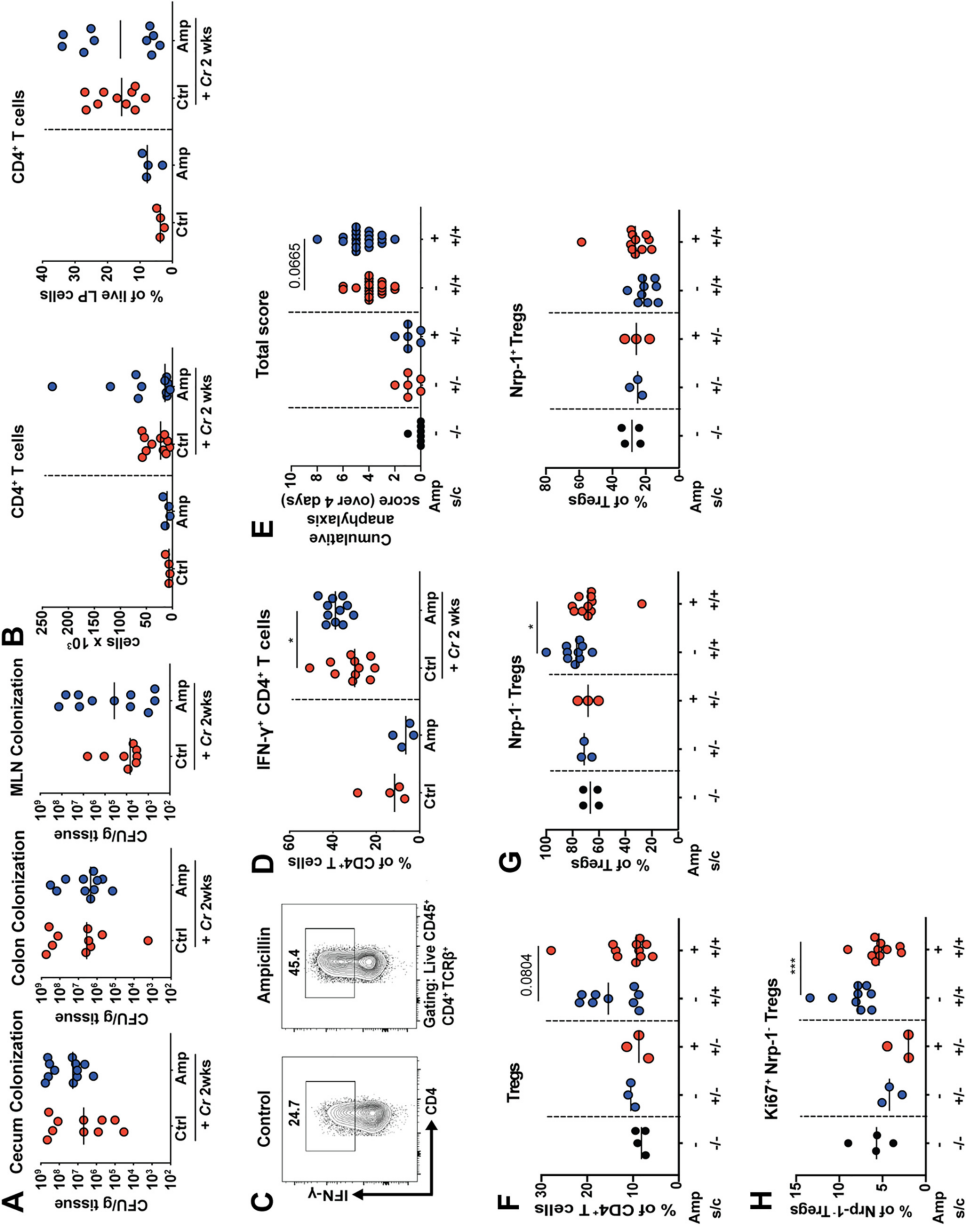
Antibiotic exposure of dams results in dysregulated Th1 responses of dysbiotic offspring to Citrobacter rodentium infection and in differential food allergy severity. (A to D) Offspring of control and ampicillin-exposed dams were intragastrically infected with Citrobacter rodentium at 7 weeks of age and sacrificed 2 weeks later. (A) C. rodentium colonization of the cecum, colon, and MLNs as determined by plating and colony counting. (B) Absolute counts per organ, and frequencies among all live cells, of CD4^+^ T cells in the colonic lamina propria as determined by flow cytometry. (C and D) Frequencies of IFN-γ^+^ CD4^+^ T cells among all CD4^+^ T cells, as assessed by *ex vivo* restimulation with PMA/ionomycin. Representative FACS plots are shown in panel C. Data in panels A to D are pooled from two independent experiments. (E to H) Offspring of control and ampicillin-exposed dams were intraperitoneally immunized twice with alum-adjuvanted ovalbumin at 5 and 7 weeks of age and challenged on four consecutive days with orally administered ovalbumin 4 weeks after the first sensitization (s/c). Control mice were sensitized with ovalbumin but mock challenged with PBS only. Additional controls were mock sensitized and mock challenged with PBS only. (E) Cumulative anaphylaxis score over 4 days. (F) Frequencies of Foxp3^+^ Tregs among all CD4^+^ T cells in MLNs. (G) Frequencies of Nrp-1^−^ cells among all Foxp3^+^ Tregs in MLNs. (H) Frequencies of Ki67^+^ cells among all Nrp-1^−^ Foxp3^+^ Tregs in MLNs. Data in panels E to H are pooled from two experiments. Horizontal lines indicate medians throughout; statistical analyses were done using the Mann-Whitney test. *, *P* < 0.05; **, *P* < 0.01; ***, *P* < 0.005. Only statistically significant differences are indicated with asterisks.

To address whether the reduction in colonic regulatory T cells that results from early-life dysbiosis affects immune responses to bacterial or allergen challenge, we first infected ampicillin-exposed or control pups with Citrobacter rodentium, a mouse-restricted Gram-negative pathogen that induces a mixed Th1/Th17 response and is a model of human colonic infections (46, 47). C. rodentium colonizes the cecum and colon and can spread to the MLNs; its ability to colonize the murine GI tract and cause disease is influenced by the status of commensal populations (12) and presence of individual species such as segmented filamentous bacteria (“*Candidatus* Savagella” species) (48). Mice were infected with C. rodentium at 7 weeks of age and assessed with respect to bacterial colonization and colonic LP T-cell responses 2 weeks later. The extents of C. rodentium colonization of the cecum, colon, and MLNs were similar in the antibiotic-exposed and control mice (Fig. 5A). The infection caused a robust influx of CD4^+^ T cells into the infected colonic LP, which also was unaffected by the early-life dysbiosis (Fig. 5B). However, *ex vivo* restimulation of T cells with phorbol myristate acetate (PMA) and ionomycin revealed that Th1, but not Th17, responses were higher in the dysbiotic mice (Fig. 5C and D; Fig. S5A and B). Next, we turned to a model of ovalbumin (OVA)-induced food allergy, for which mice were first sensitized intraperitoneally with alum-adjuvanted ovalbumin and then challenged orally with ovalbumin on four consecutive days. Mice were scored daily for the development of anaphylactic symptoms and assessed with respect to their splenic Th2 cytokine production at the study endpoint. The antibiotic-exposed pups showed trends toward higher anaphylaxis scores (repeated scratching around the nose and mouth, puffy eyes and nose, and reduced activity [Fig. 5E]) and expressed somewhat higher levels of splenic interleukin 5 (IL-5) and IL-13 as assessed by enzyme-linked immunosorbent assay (ELISA) and reverse transcription-quantitative PCR (qRT-PCR) after *ex vivo* restimulation with ovalbumin (Fig. S5C and D). Ovalbumin sensitization and challenge resulted in an increase in the frequencies of Tregs, especially of Nrp-1^−^ Tregs, in the MLNs; however, the increased frequency of Tregs was reduced in antibiotic-exposed mice (Fig. 5F and G). A small fraction of Nrp-1^−^ Tregs in MLNs were positive for Ki67, and this fraction was reduced in antibiotic-exposed mice (Fig. 5H). The combined results indicate that early-life dysbiosis of the small intestine and colon affects T-cell responses to infectious challenge in the colon and causes somewhat enhanced systemic responses to an orally administered antigen in a food allergy model.

10.1128/mBio.03335-20.5 FIG S5 Effects of perinatal antibiotic exposure on T-cell responses to bacterial infection and allergen challenge. (A and B) Control and ampicillin-pretreated mice were infected intragastrically with a single inoculum of Citrobacter rodentium (1 × 10^8^ CFU) at 7 weeks of age and analyzed 2 weeks later with respect to their colonic T-cell responses. TNF-α^+^ (A) and IL-17^+^ (B) cells among all colonic CD4^+^ T cells are shown. (C and D) Control and ampicillin-pretreated mice were intraperitoneally immunized twice with alum-adjuvanted ovalbumin at 5 and 7 weeks of age and challenged on four consecutive days with orally administered ovalbumin 4 weeks after the first sensitization (s/c). Control mice were sensitized with ovalbumin but mock challenged with PBS only. Additional controls were mock sensitized and mock challenged with PBS only. Splenic IL-5 and IL-13 production after 4 days of *ex vivo* restimulation of splenocytes with ovalbumin was assessed by ELISA (C) and qRT-PCR (also performed for IL-4 [D]). Data in panels A and B are pooled from two experiments; data in panels C and D are from one of two independent experiments. Horizontal lines indicate medians throughout; statistical analyses used the Mann-Whitney test. *, *P* < 0.05. Download FIG S5, TIF file, 2.2 MB. Copyright © 2021 Zhang et al. 2021 Zhang et al. This content is distributed under the terms of the Creative Commons Attribution 4.0 International license.

## DISCUSSION

We show here that the exposure of lactating dams and pups to a broad-spectrum β-lactam antibiotic causes persistent dysbiosis and a reduction of colonic CD4^+^ T cells and, in particular, of colonic Nrp-1^−^ RORγt^+^ Tregs. These effects were specific to the colon and not observed in the lungs, upper GI tract, or spleen. Myeloid and granulocyte populations did not differ in the offspring, suggesting that T cells, but not other leukocyte lineages, are selectively dependent on a healthy, diverse gut microbiota. Only extrathymically induced Nrp-1^−^ RORγt^+^ Tregs, and not thymus-derived Nrp-1^+^ Tregs (which we, like others investigators, for lack of other markers distinguish largely based on the surface marker Nrp-1 [43]), depend on an unperturbed gut microbiota. Previous work, mostly conducted using germfree mice, has documented important roles of intestinal microbes in colonic Treg induction (49). In addition to other deficits in the development of the gut-associated lymphoid tissues (GALT), including reduced number and size of Peyer’s patches and MLNs, germfree mice have reduced frequencies and absolute numbers of colonic, but not small intestinal, Tregs, which can be reversed by the introduction of an altered Schaedler flora (50) or a mixture of *Clostridium* species (49). While the ability to induce colonic Tregs was initially proposed to be a trait of Gram-positive bacteria (49), it is now clear that both Gram-positive and Gram-negative GI tract resident bacterial species can effectively induce Tregs with highly suppressive activity in both the colon and other gastrointestinal tissues. Examples include Helicobacter pylori colonizing the stomach (51), Helicobacter hepaticus colonizing the cecum (52, 53), and a group of 12 human commensal species (from both *Bacteroidetes* and *Firmicutes*, including lactobacilli) which in a monocolonization model of germfree mice all were able to induce Nrp-1^−^ RORγt^+^ Tregs (44). The order *Bacteroidales*, especially the genus *Bacteroides*, was particularly rich in species able to induce colonic Tregs in a large-scale screen designed to identify bacterial species among human fecal microbiota that in monocolonization models of germfree mice exhibit that trait (54). Our observation that the genus *Bacteroides* is underrepresented in ampicillin-impacted communities is consistent with the prior studies.

Colonic Nrp-1^−^ RORγt^+^ Treg differentiation is a multistep process that is initiated in the MLNs by a tissue-derived migratory CD103^+^ DC population that drives Foxp3 and gut homing receptor expression in naive T cells (55–57). MLN DCs cross talk with stromal cells in the MLNs, and both compartments are key cellular sources of tolerogenic molecules, including retinoic acid, transforming growth factor β (TGF-β), and BMP2, which are all known to drive Treg differentiation (56–59). While migratory CD103^+^ DCs, presenting luminal antigens they have encountered in the gastrointestinal tract, appear to contribute more effectively to overall Treg generation in the MLNs, MLN resident DCs also have Treg-inducing capacity, which appears to be imprinted by stromal cells in a microbiota-dependent manner in the neonatal period and mediated by the TGF-β superfamily member BMP2 (59). Tregs generated *de novo* in the MLNs subsequently migrate to and expand within the intestinal lamina propria (45), and these processes are supported by microbiota and their metabolic products (60, 61). Our data are consistent with the notion that a diverse and healthy microbiota is required at the earliest steps in Treg priming that happen in the MLNs, based on three observations: (i) DCs isolated from antibiotic-impacted MLNs are less capable of Treg induction *in vitro* than DCs from control mice; (ii) ovalbumin-induced Treg -and especially Nrp-1^−^ RORγt^+^ Treg- populations are reduced in the MLNs of antibiotic-impacted, ovalbumin-challenged mice; and (iii) the fraction of proliferating Tregs is reduced in the MLNs, but not the colonic LP, of antibiotic-impacted mice. The last observation suggests that once *de novo*-induced Tregs have successfully trafficked to the colonic LP, they have become largely independent of microbial or other environmental cues; our results therefore are consistent with the concept that neonatal tolerogenic imprinting affects extrathymic Treg generation at the priming stage in MLNs.

Both Nrp-1^−^ (p)Tregs and thymus-derived Tregs, and subsets of both populations, have alternatively been implicated in generating and maintaining immune tolerance of intestinal commensals and in suppressing excessive effector T-cell responses to pathogens (62–65). However, the current consensus is that extrathymically induced Tregs are the main drivers of tolerance to food and commensal antigens and essential for intestinal homeostasis (44, 45, 66–68). Colonic Treg induction has been attributed to the ability of commensals to produce the short-chain fatty acids (SCFAs) butyrate, acetate, and propionate (68), which—when administered in purified form—are sufficient to drive colonic Treg differentiation and alleviate immunopathology in several Treg-controlled disease models (69). In our model of antibiotic exposure leading to dysbiosis, we observed the most robust Treg shifts in neuropilin-1^−^ Tregs that express RORγt. Several studies have documented the suppressive activity of RORγt^+^ Foxp3^+^ Tregs, and the critical role of RORγt expression in Foxp3^+^ Tregs, in models of type I and type II immunity. The specific ablation of RORγt in Foxp3^+^ Tregs was sufficient to exacerbate 2,4,6-Trinitrobenzenesulfonic acid (TNBS)-induced colitis driven by pathogenic Th1 and Th17 cells (44) but also the Th2-driven pathology of oxazolone-induced colitis (70). Th2-driven helminth control was improved in mice lacking RORγt specifically in Foxp3^+^ Tregs (70). Furthermore, the success of experimental therapy of Th2-driven food allergy with two different bacterial consortia, consisting of either *Clostridiales* or *Bacteroidales* species, required RORγt expression in Foxp3^+^ Tregs (71). These cells selectively suppressed specific Th17 responses in a Helicobacter hepaticus-driven model of colonic RORγt^+^ Foxp3^+^ Treg generation and function (52). We observe robust effects of the early-life dysbiosis on Th1 (but not Th17) responses to Citrobacter rodentium challenge and modest effects on Th2 responses to orally administered ovalbumin. The latter result parallels earlier studies that have reported more severe allergic airway inflammation and atopic dermatitis in mice subjected to early life therapeutic antibiotic exposure (72–74). The combined data from our model support several previous studies that have implicated colonic RORγt^+^ Tregs in the rather general (nonselective) suppression of various types of T-helper (Th1, Th2, and Th17) responses (49, 68). This feature sets these cells apart from T-bet-expressing Tregs, which exclusively arise in Th1-polarized settings and are functionally specialized to suppress Th1 but not other T-helper cells (75, 76).

Our metagenomics analyses have revealed that the dams’ dysbiotic microbiome invariably clusters with their offspring’s microbiomes. Two alternative mechanisms can in principle result in the persistent shifts in the composition of the gut microbiota and the co-occurring changes in the Treg populations that we observed in the offspring. On the one hand, antibiotics in an exposed dam’s milk (77) may directly select colonic microbes as the microbiota population structure is gradually assembling in the suckling mice. Alternatively, the pups acquire their mother’s antibiotic‐impacted dysbiotic microbiota during the critical neonatal window, with little chance to adjust its composition once that dysbiotic community has stabilized. The fact that cohousing with normal control mice fails to reverse the Treg phenotype indicates the importance of these early-life effects, which are consistent with our prior studies (28).

Our model of transmaternal antibiotic exposure recapitulates many aspects of perinatal antibiotic exposure in human children, including intrapartum antibiotic prophylaxis (IAP), i.e., the common practice of administering high doses of intravenous antibiotics (penicillin or ampicillin) to prevent early‐onset neonatal group B streptococcal (GBS) disease. IAP has been identified as a major cause of disrupted microbiota transmission from mother to neonate in a UK study examining 596 full-term babies (36), with reduced intergenerational transmission of *Bacteroides* species being particularly prevalent as a consequence of IAP (36) and/or C-section delivery (20). In the former study, children born by Cesarean section or exposed to IAP were more likely to be colonized by nosocomial pathogens (including *Enterococcus*, *Enterobacter*, and *Klebsiella* species) than controls (36); IAP also has been independently linked to early-onset neonatal sepsis (35). Experimental evidence suggests that both transmaternal and direct antibiotic exposure of neonatal mice compromises CD8^+^ T-cell responses to Escherichia coli K-12 or Klebsiella pneumoniae-induced neonatal sepsis (37) and to viral pathogens such as vaccinia virus (34) or lymphocytic choriomeningitis virus (LCMV) and influenza virus (21).

Our results provide experimental evidence for the notion that the development not only of antiviral or antibacterial immunity, as shown in the above-mentioned studies, but also of immune regulation is compromised by an antibiotic-impacted dysbiotic microbiota in early life. Perinatal dysbiosis persists into adulthood and is not readily reversed by exposure to an unperturbed microbiota after weaning. We show that early-life dysbiosis is linked to dendritic cell dysfunction and results in dysregulated effector T-cell responses to an intestinal pathogen and in more severe food allergy.

## MATERIALS AND METHODS

### Animal experimentation.

C57BL/6 mice were purchased from Janvier and bred and maintained under specific-pathogen-free conditions in certified animal facilities at the University of Zurich. For antibiotic exposure, ampicillin was dissolved in distilled deionized water at a concentration of 0.333 mg/ml, and mice were exposed to ampicillin through their mothers’ milk as described previously (77, 78); alternatively, neonatal mice were treated by oral gavage on five consecutive days with ampicillin for a daily dose of 100 mg/kg of body weight. For use in fecal microbiota transplantation, fecal pellets were collected and immediately frozen at −80°C. Approximately 25 mg of fecal pellets of two control mice and two ampicillin-exposed mice each were homogenized into 500 μl of beef broth medium (10.0 g of beef extract, 10.0 g of peptone, and 5.0 g of NaCl in 1.0 liter of water, pH 7.2) to produce fecal suspensions. The donor fecal samples were not pooled, and a fecal suspension was prepared for each donor mouse. Fifty microliters of each fecal suspension was administered by gavage to six recipient germfree mice, and the mice were maintained for 4 weeks before sacrifice (total *n* = 24 mice). For cohousing experiments, antibiotic-pretreated and -naive female mice were pooled at weaning and maintained together for 4 weeks until the study endpoint.

### C. rodentium infection.

The nalidixic acid (NAL)-resistant Citrobacter rodentium strain ICC169 was grown overnight at 37°C in Luria broth (LB) supplemented with NAL (50 μg/ml; Sigma). Mice were infected with a single oral dose of 1 × 10^8^ bacteria at 7 weeks of age and maintained for 14 days. To assess C. rodentium colonization, cecal, colonic, and mesenteric lymph node (MLN) tissues were homogenized in phosphate-buffered saline (PBS), diluted, and inoculated onto LB plates supplemented with NAL (LB-NAL). Colonies were counted after 18 h of aerobic culture at 37°C.

### Ovalbumin-induced food allergy.

For ovalbumin (OVA)-induced food allergy, C57BL/6 mice were sensitized twice intraperitoneally (i.p.) with 50 μg of OVA (Sigma; A5503-5G) emulsified in aluminum hydroxide (Imject alum, 77161; Thermo Scientific) on days 0 and 14, followed by challenge via oral gavage on days 28, 29, 30, and 31 with 60 mg of OVA. Signs were scored for 40 min after each challenge, with scores indicating the following: 0, no sign of reaction; 1, repetitive scratching and rubbing around the nose/mouth and head and ear canal digging with hind legs; 2, decreased activity with an increased respiratory rate, pilar erecti, and/or puffing around the eyes and/or mouth; 3, labored respiration and cyanosis around the mouth and tail and/or periods of motionless for more than 1 min or lying prone on stomach; 4, slight or no activity after prodding/whisker stimuli or tremors and convulsion; and 5, death. Cumulative scores over 4 days were calculated by adding all four individual scores per mouse. Mice were sacrificed by CO_2_ inhalation after the last challenge, and blood and tissue samples were collected. For splenic antigen-specific Th2 cytokine ELISAs, spleens were pushed through a 40-μm cell strainer and washed with PBS prior to red blood cell lysis. Splenocytes were seeded into 96-well plates in RPMI 1640 medium (Gibco 21875-034 plus fetal calf serum [FCS] and penicillin-streptomycin) supplemented with 200 μg/ml of OVA protein. After 4 days in culture, supernatants were collected and cytokines were quantified by IL-5 (88-7054-88) and IL-13 (88-7137-88) ELISA, according to the manufacturer’s instructions (eBioscience).

### Leukocyte isolation.

For lamina propria (LP) leukocyte isolation, gastrointestinal tissues were opened longitudinally, washed, and cut into sections that were incubated for 1 h in Hanks’ balanced salt solution with 10% FCS and 5 mM EDTA at 37°C to remove epithelial cells. The remaining tissue was digested at 37°C for 50 min in a shaking incubator with 15 mM HEPES, 500 U/ml of type IV collagenase (Sigma-Aldrich), and 0.05 mg ml^−1^ of DNase I in supplemented RPMI 1640 medium. Cells were then layered onto a 40/80% Percoll gradient and centrifuged, and the interface was washed in PBS with 0.5% bovine serum albumin (BSA). Lymph node cell suspensions were prepared by digestion in 500 U/ml of type IV collagenase in RPMI 1640 for 15 min followed by passage through a cell strainer using a syringe plunger. Lung cell suspensions were prepared by perfusing the lung with PBS followed by passage through a cell strainer.

### Flow cytometry, T-cell restimulation, and cell counting.

Cells were stained with a fixable viability dye and a combination of the following antibodies: anti-mouse CD45 (clone 30-F11), CD11c (N418), major histocompatibility complex class II (MHC-II; M5/114.15.2), F4/80 (BM8), CD103 (M2E7), CD11b (M1/70), CD3e (145-2C11), CD4 (RM4-5), CD8 (53-6.7), T-cell receptor β (TCRβ; H57-597), neuropilin-1 (3E12), or an IgG isotype control (all from BioLegend). Fc block (anti-CD16/CD32; Affymetrix) was included to minimize nonspecific antibody binding. For intracellular cytokine staining, cells were incubated at 37°C for 3.5 h in complete Iscove's Modified Dulbecco's Medium (IMDM) supplemented with 0.1 μM phorbol 12-myristate 13-acetate, 1 μM ionomycin, 1:1,000 brefeldin A (eBioscience), and GolgiStop solutions (BD Biosciences) at 37°C in a humidified incubator with 5% CO_2_. Following surface staining, cells were fixed and permeabilized with the Cytofix/Cytoperm fixation/permeabilization solution kit (CC-kit; BD Biosciences) according to the manufacturer’s instructions. Cells were stained for 50 min with antibodies to IL-17A (TC11-18H10.1), gamma interferon (IFN-γ; XMG1.2), and tumor necrosis factor alpha (TNF-α; MP6-XT22). For intranuclear staining of transcription factors, cells were fixed and permeabilized with the Foxp3/transcription factor staining buffer set (eBioscience) after surface staining according to the manufacturer’s instructions. Cells were stained for 50 min with antibodies to FoxP3 (FJK-16s) and RORγt (B2D) from Invitrogen. Samples were acquired on an LSRII Fortessa (BD Biosciences) and analyzed using FlowJo software.

### Dendritic cell/T-cell cocultures.

For DC/T-cell cocultures, CD11c^+^ DCs were isolated by positive selection from MLN single-cell suspensions using CD11c ultrapure mouse microbeads (Miltenyi Biotech). Naive CD4^+^ T cells were isolated by negative selection from splenic single-cell suspensions using the MagCellect mouse naive CD4^+^ T-cell isolation kit (R&D Systems). DCs and T cells were cocultured at 1:5 ratios (20,000 DCs and 100,000 T cells) in a round-bottom 96-well plate for 72 h in the presence of anti-CD3 agonistic antibody (BD Bioscience) at a 1-μg/ml final concentration with 5 ng/ml of TGF-β1 and 10 ng/ml of IL-2 (both from R&D Systems). Foxp3^+^ Tregs were stained as described above, and samples were acquired on an LSRII Fortessa (BD Biosciences) and analyzed using FlowJo software.

### 16S rRNA analyses.

Intestinal contents and fecal pellets were collected from mice and frozen in liquid nitrogen when collected and stored at –80°C. DNA extraction was completed using a DNeasy PowerSoil HTP 96 kit (Qiagen) according to the manufacturer’s protocol. The V4 region of the 16S rRNA was amplified using the 515F/806R primer pair with barcodes located in the forward primer as described previously (79). PCRs were completed in triplicate for each sample, and amplicon DNA concentrations were quantified using a PicoGreen assay (Invitrogen). Amplicons were pooled at equal quantities of DNA, and PCR cleanup was done using a QIAquick PCR purification kit (Qiagen). DNA was quantified using the QuBit double-stranded DNA (dsDNA) high-sensitivity assay (Invitrogen), and the amplicon library was pooled at equimolar concentrations. The 16S rRNA library was sequenced on an Illumina MiSeq 2 × 150-bp platform at New York University Langone Genome Technology Center. Sequence reads were processed using the QIIME2 pipeline (80); reads were filtered and trimmed and an ASV table generated using the DADA2 pipeline (81). Taxonomy was assigned using SILVA 138 (released December 2019). Alpha (Shannon and observed ASVs) and beta diversity (unweighted and weighted UniFrac) analyses, rarefied at 5,000 reads, were performed using the QIIME2 pipeline, and plots were generated in R using ggPlot2. Taxonomic abundance plots were generated using the Phyloseq package, and differential abundance plots and heat maps were generated using the DeSeq2 and Complex Heatmap packages in R (82, 83).

### Statistical analysis.

Statistical analysis was performed with Prism 6.0 (GraphPad Software). The nonparametric Mann-Whitney U test was used for all direct statistical comparisons between two groups. Multiple-group comparisons were performed by one-way analysis of variance (ANOVA) followed by Holm-Sidak’s multiple-comparisons correction. Differences were considered statistically significant when the *P* value was <0.05. In figures, *P* values are indicated as follows: *, *P* < 0.05; **, *P* < 0.01; ***, *P* < 0.001; and ****, *P* < 0.0001. Differentially abundant ASVs were determined by contrasting the relevant treatment groups and selecting for ASVs that were significant using a Benjamini-Hochberg false-discovery rate (FDR; *P* value) of <0.01.

### Ethics statement.

All animal experimentation at the University of Zurich was reviewed and approved by the Zurich Cantonal Veterinary Office (licenses ZH140/2017 and ZH086/2020 to A.M.) and adhered to the rules and regulations of the Swiss National Veterinary Office. All mouse experiments at NYU Langone were approved by the New York University Langone Institutional Animal Care and Use Committee (IACUC protocol IA16-00785) and complied with federal and institutional regulations.

### Data availability.

The 16S sequencing data are available through EBI with accession number PRJEB42154 or via Qiita with accession code Study 13509.
