# Supplementary figures and images for: An Antibiotic-Impacted Microbiota Compromises the Development of Colonic Regulatory T Cells and Predisposes to Dysregulated Immune Responses
Source: mBio. 2021 Feb 2;12(1):e03335-20. doi: 10.1128/mBio.03335-20 (PMC7858066; doi:10.1128/mBio.03335-20)

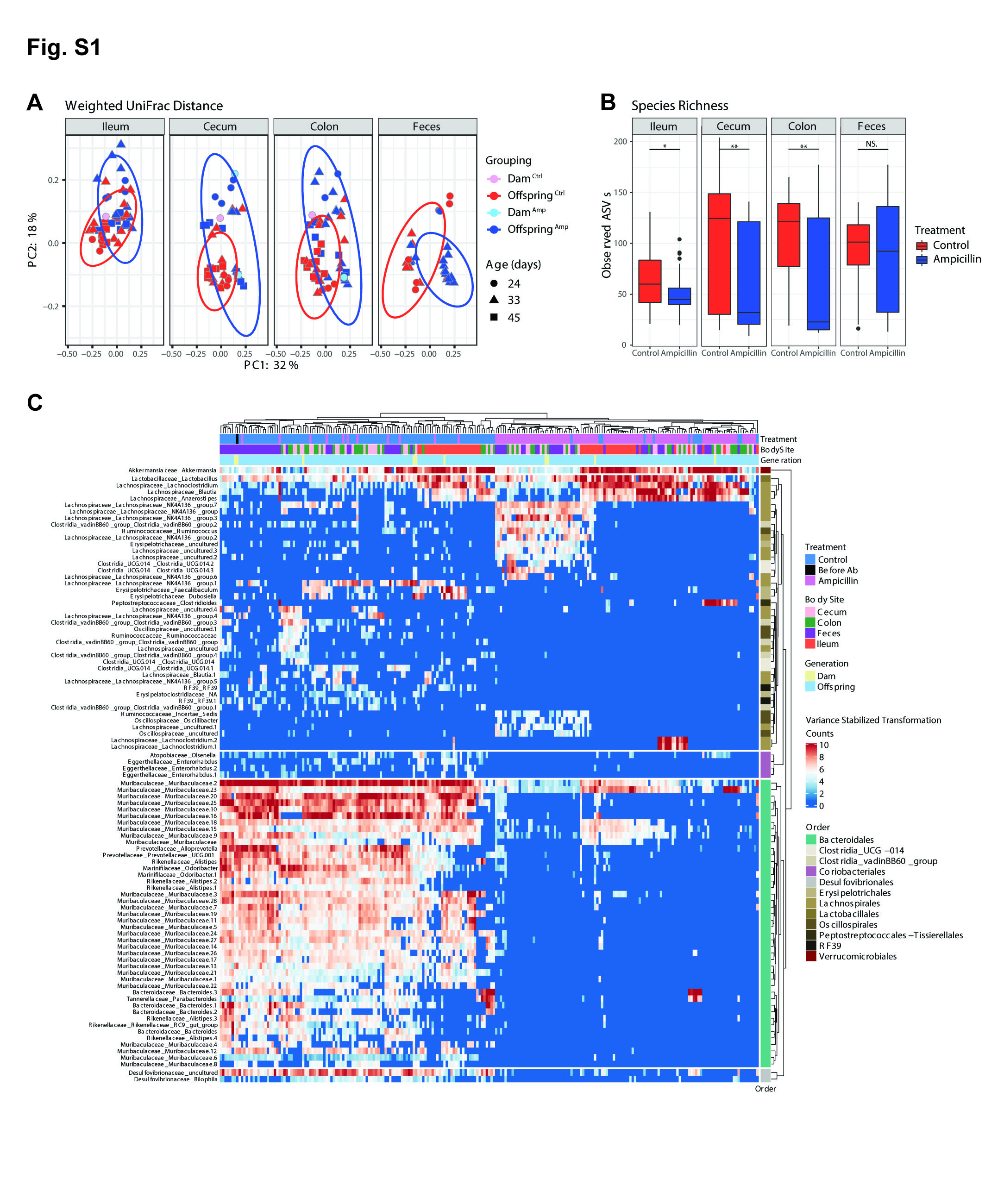

Supplement: FIG S1 [file mBio.03335-20-sf001.jpg]

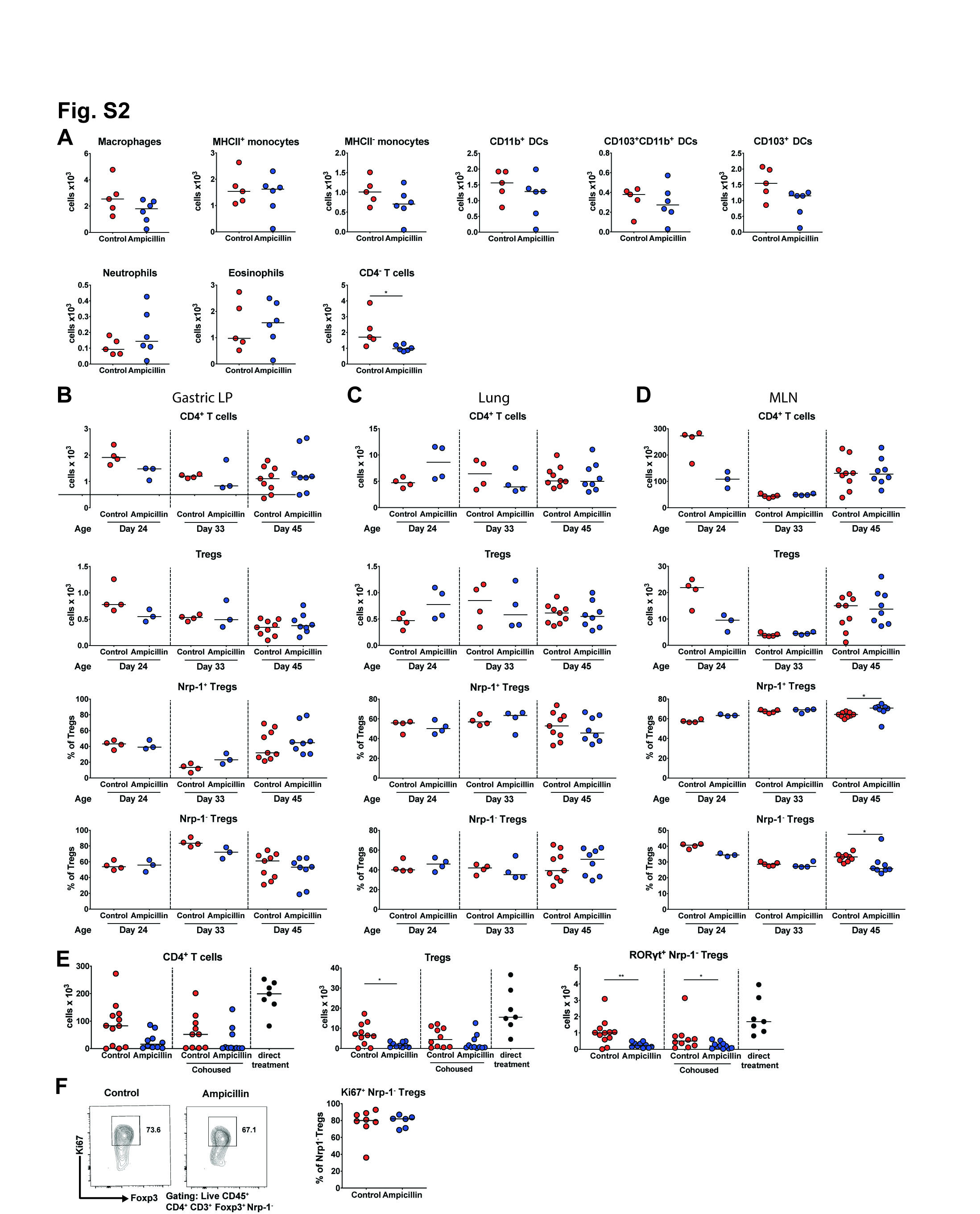

Supplement: FIG S2 [file mBio.03335-20-sf002.jpg]

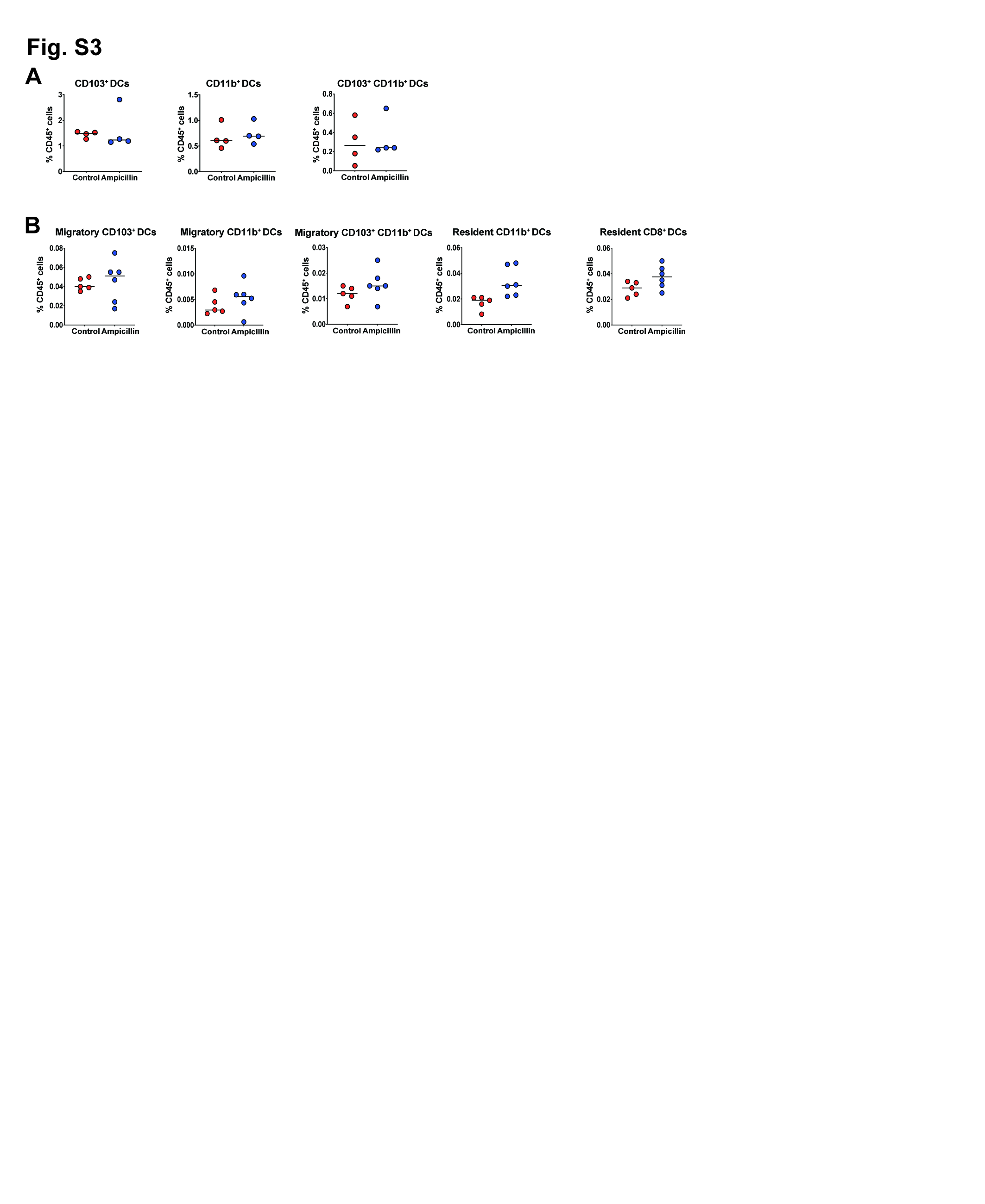

Supplement: FIG S3 [file mBio.03335-20-sf003.tif]

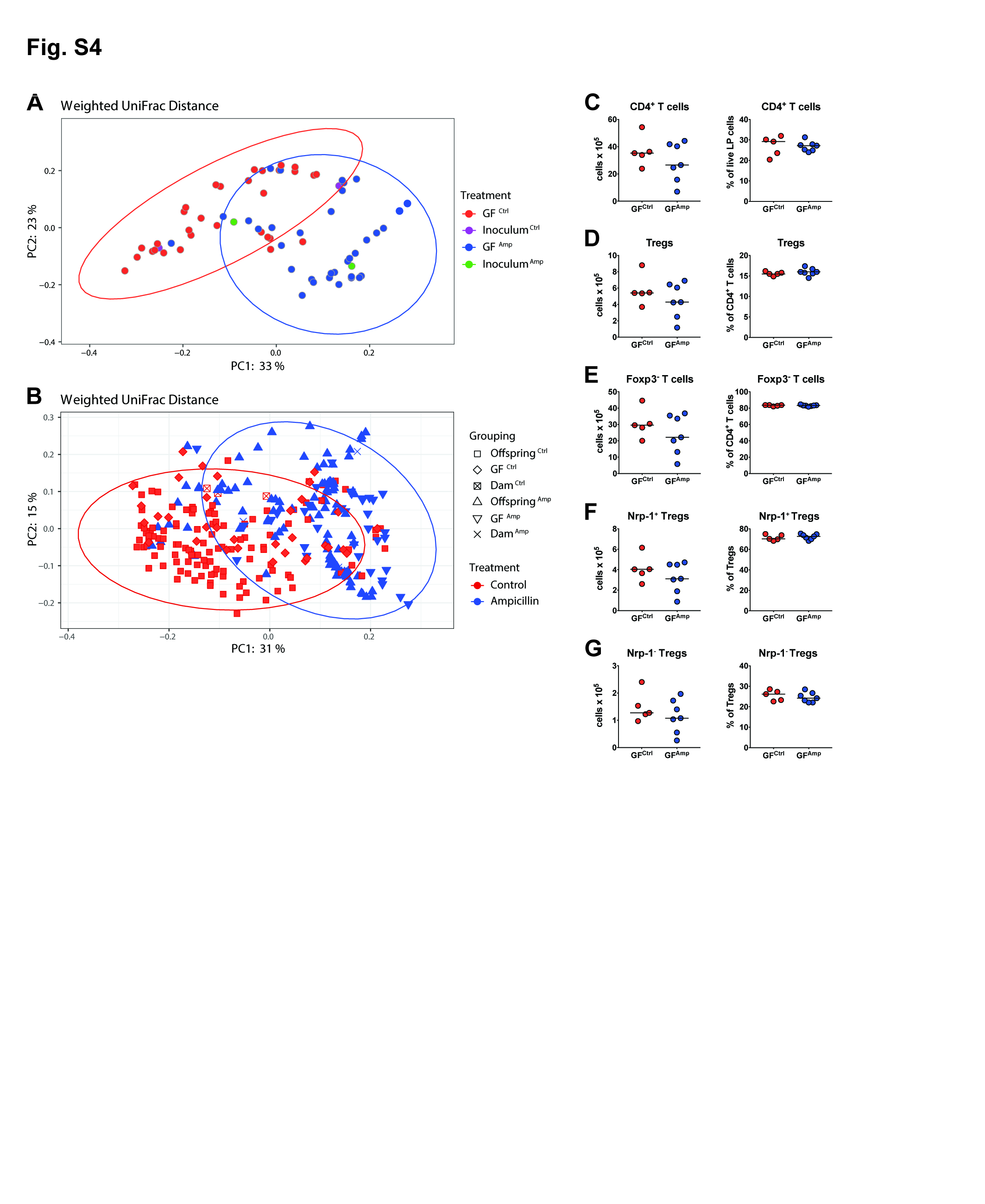

Supplement: FIG S4 [file mBio.03335-20-sf004.tif]

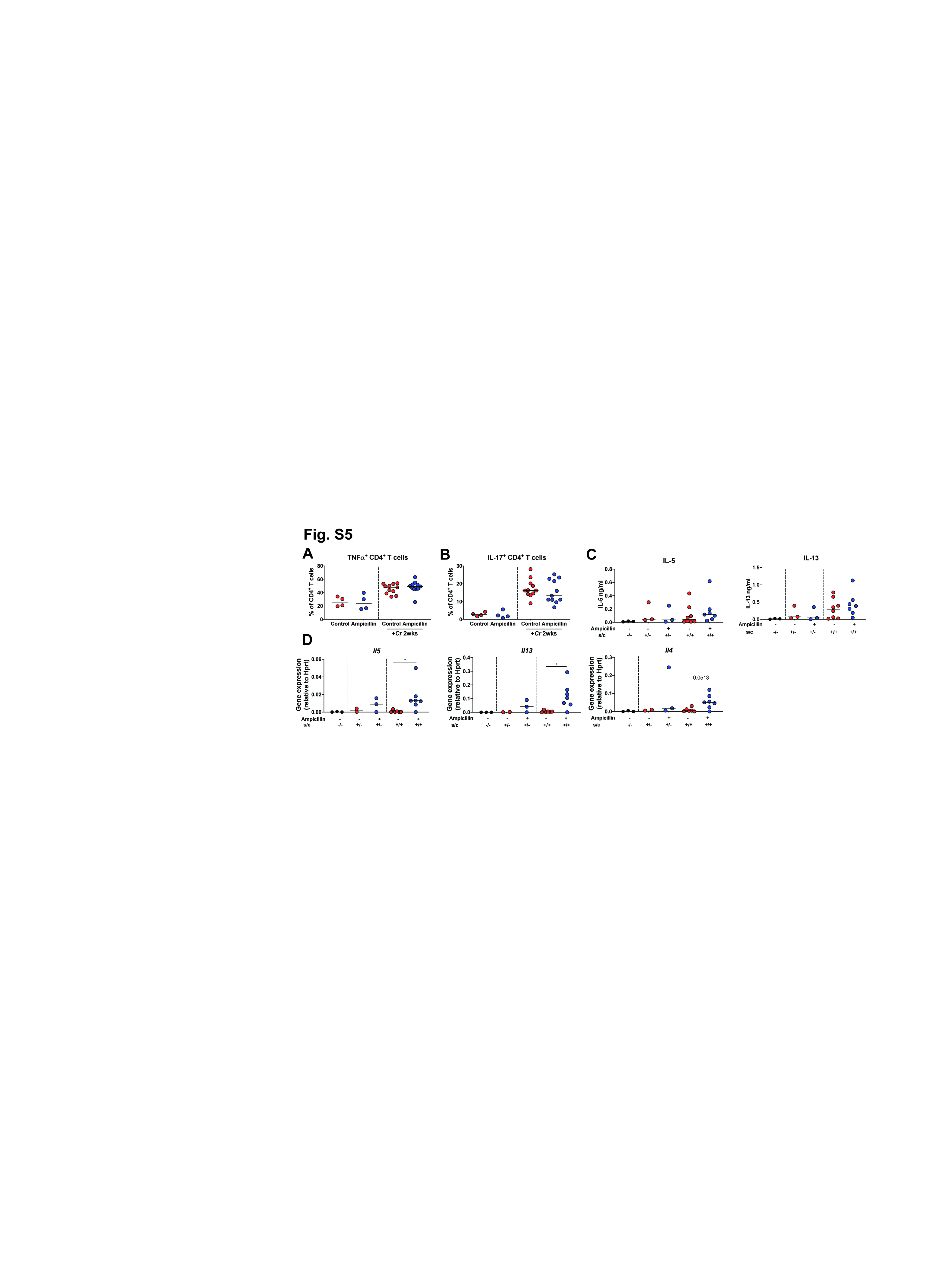

Supplement: FIG S5 [file mBio.03335-20-sf005.tif]
